# Faecal Diagnostic Biomarkers for Colorectal Cancer

**DOI:** 10.3390/cancers13215568

**Published:** 2021-11-07

**Authors:** Andrea Cruz, Carla M. Carvalho, Alexandra Cunha, Anais Crespo, Águeda Iglesias, Laura García-Nimo, Paulo P. Freitas, Joaquín Cubiella

**Affiliations:** 1 International Iberian Nanotechnology Laboratory, 4715-330 Braga, Portugal; alexandra.cunha@inl.int (A.C.); paulo.freitas@inl.int (P.P.F.); 2 Servicio de Aparato Digestivo, Complexo Hospitalario Universitario de Ourense, Instituto de Investigación Sanitaria Galicia Sur, CIBEREHD, 32005 Ourense, Spain; Anais.Crespo.Lois@sergas.es (A.C.); Agueda.Iglesias.Gomez@sergas.es (Á.I.); Joaquin.Cubiella.Fernandez@sergas.es (J.C.); 3 Servicio de Análisis Clínicos, Complexo Hospitalario Universitario de Ourense, Instituto de Investigación Sanitaria Galicia Sur, 32005 Ourense, Spain; Laura.Garcia.Nimo@sergas.es

**Keywords:** colorectal cancer, advanced adenoma, diagnosis, biomarkers, faecal haemoglobin, M2-PK

## Abstract

**Simple Summary:**

Colorectal cancer (CRC) is a significant public health problem, being a major cause of cancer death worldwide. Hence, the identification of biomarkers able to support CRC detection is crucial. This work analyses a panel of six biomarkers, namely interleukin-6 (IL-6), tumour necrosis factor-alpha (TNF-α), matrix metalloproteinase (MMP)-2, MMP-9, haemoglobin (Hb) and M2-pyruvate kinase (M2-PK), in stool samples from patients with CRC, advanced adenomas, other lesions and healthy individuals. Our results indicate that the levels of Hb and M2-PK were increased in CRC patients in comparison to the controls. Moreover, the combination of these biomarkers increased the specificity or sensitivity for CRC detection and thus present potential for diagnosis of CRC.

**Abstract:**

Background: Colorectal cancer (CRC) is a major cause of cancer-related death worldwide. Cancer progression, including invasion and metastasis, is a major cause of death among CRC patients. Current methods for CRC screening commonly consist of a combination of faecal immunochemical test (FIT) for stool occult blood detection and invasive procedures such as colonoscopy. Considering the slow progression of CRC, and that symptoms usually emerge at advanced stages, its early diagnostic can limit cancer’s spread and provide a successful treatment. Biomarkers have a high potential for the diagnosis of CRC in either blood or stool samples. Methods: In this study, we analysed the diagnostic value of six different biomarkers in stool samples of patients with CRC, advanced adenomas, other lesions and healthy individuals. We have also assessed the overall performance of the combination of these biomarkers for CRC detection. Results: The results indicate that haemoglobin (Hb) and M2-pyruvate kinase (M2-PK) levels were increased in CRC patients in comparison to the controls. Conversely, the concentrations of matrix metalloproteinase (MMP)-2, MMP-9, and tumour necrosis factor-alpha (TNF-α) were not significantly different between the tested groups. Conclusion: The combination of FIT-Hb with the M2-PK levels increased the specificity or sensitivity for CRC detection and thus present potential as faecal diagnostic biomarkers for CRC.

## 1. Introduction

Colorectal cancer (CRC) is a leading cause of cancer-associated morbidity and mortality worldwide, being the third most frequently diagnosed cancer among adults and one of the leading causes of cancer-related death [1,2]. The International Agency for Research on Cancer (IARC) estimates that the global incidence of CRC for 2018 was 1,801,000 new cases and 861,700 related deaths, and it is anticipated to increase by 60% in 2030, with 2.2 million new cases and 1.1 million deaths [2,3]. Most CRC cases occur sporadically and are characterised by a slow progression that involves benign polyps that gradually evolve to invasive and metastasising advanced neoplasms [4,5]. This slow progression makes CRC one of the most preventable diseases; however, it entirely depends on its early detection, which is challenging, as clinical symptoms usually emerge only when this disease is already advanced [4].

Colonoscopy is still considered the gold standard diagnostic technique for CRC screening since it can be combined with the simultaneous removal of detected polyps and therefore work as a diagnostic and preventive procedure [6]. Nevertheless, colonoscopy is an expensive and invasive procedure, and thus its acceptance among patients is low [4,7,8]. Regarding non-invasive CRC screening methods, the most commonly used is based on the detection of hidden blood in the stool, namely the guaiac faecal occult blood test (gFOBT) and the faecal immunochemical test for haemoglobin (FIT-Hb). The gFOBT measures the heme (non-protein) part of the Hb, while FIT-Hb for the globin (protein) part of Hb [4,7,9]. Since gFOBT can detect any source of blood, it cannot distinguish bleeding between the upper and lower gastrointestinal tract. Additionally, it lacks sensitivity and is affected by drugs or diet [10]. Therefore, this method has been replaced by the FIT-Hb, which can detect and quantify faecal Hb and presents many advantages when compared to gFOBT, such as higher sensitivity for CRC and advanced adenomas; it only requires one stool sample and is more cost-effective [9,11,12]. Nevertheless, both these techniques require a confirmatory colonoscopy [8,13].

Despite the opportunity for CRC early diagnosis due to its slow progression, around 25% of the patients are diagnosed at stage IV with distant metastasis and a 5-year survival rate under 10% [5]. Thus, there is an urgent need for a non-invasive, easy, specific, and accurate screening method to safely diagnose patients with CRC.

Biomarkers have been emerging as tools for the early detection and prognostic stratification, surveillance and therapy selection for several diseases, namely CRC [14,15]. A biomarker is defined as a measurable biologically plausible parameter, usually being an indicator of an underlying disease mechanism [16]. An ideal biomarker should be easily measured, quantifiable, reliable, reproducible, highly specific and sensitive, and able to differentiate between different risk-based populations [9]. This should be ideally achieved with a non-invasive and inexpensive method using easily available biological samples, particularly saliva, urine, or stool [4,9,17].

Biomarkers present a high potential for clinical application and can be used for diagnostic, predictive, or prognostic purposes [15]. Diagnostic biomarkers are able to detect or suggest the presence of a patient’s disease or condition, while predictive biomarkers are used to indicate the response to a specific treatment and to guide the decision-making process. The prognostic biomarkers give information about the patient’s overall cancer outcome [5]. The identification of biomarkers that can support CRC early detection or monitoring would enable the implementation of a potentially curable treatment before the spread of cancer [9,17].

Among all biomarkers, there are a few that have revealed a higher potential for CRC detection [4]. These include the M2-pyruvate kinase (M2-PK) that is expressed during cancer development, playing an important role in neoplastic growth and glycolysis during carcinogenesis and thus has been described as an indicator for a wide range of cancers, including CRC [18,19,20]. Other biomarkers, including matrix metalloproteinases (MMPs) such as MMP-2 and MMP-9, have also been investigated, since they are related to the pathology of cancers, including but not limited to invasion, metastasis, and angiogenesis [21]. These are a family of zinc-dependent endopeptidases, secreted by various cell types, such as fibroblasts, inflammatory mesenchymal, and tumour cells [22,23]. It has also been reported that cancer-associated inflammation is an important indicator of disease progression and survival in CRC [24]. As inflammatory cytokines, interleukin-6 (IL-6) and tumour necrosis factor-alpha (TNF-α) are key players in the regulation of inflammation [25,26]. These cytokines are characterised by a broad spectrum of functions, including cytotoxic and cytostatic effects against cancer cells, and therefore show potential as CRC indicators [25,26,27,28].

In this work, we analysed the diagnostic value of a panel of biomarkers, namely Hb, M2-PK, MMP-2, MMP-9, IL-6, and TNF-α for CRC detection in stool samples from patients with CRC, advanced adenoma, and other lesions, as well as healthy patients as controls.

## 2. Materials and Methods

Patient selection and sample collection. Overall, 216 patients (male/female: 117/99; median age: 70, range 17–88 years) referred to our specialist colorectal unit in the Complexo Hospitalario Universitario de Ourense were enrolled in the study, who all underwent colonoscopy. Based on the colonoscopy and histological results, patients were allocated to four groups: negative colonoscopy (controls), other lesions (non-advanced adenoma, significative, and non-significative colonic lesion), advanced adenoma, and CRC. The study protocol was approved by the Ethical Research Committee of Galicia (2019/135, 02/04/2019). All subjects provided written and informed consent to participate.

Biomarkers Analysis: FIT for Hb detection. FIT-Hb was used for detecting occult Hb in red blood cells on stools, using the OC-SENSOR device (Eiken Chemical Co., Tokyo, Japan). Stool samples were collected using the OC-Auto Sampling Bottle, which is a grooved probe that holds approximately 10 mg of faeces and contains 2 mL of Hb stabilisation buffer. Prior to analysis, the auto-analyser was calibrated with a standard curve of known Hb concentration. Additionally, low and high Hb concentration quality control samples were run with each batch to ensure machine accuracy (as per manufacturer instructions and following the hospital laboratory guidelines). The analytical range of the method is 20–1000 ng/mL.

Enzyme-Linked Immunosorbent Assay (ELISA):

Sample preparation. Stool samples were collected in a specimen tube at home, delivered at the hospital, and immediately aliquoted in 2 mL eppendorf and stored at −80 °C until processed. Before biomarkers’ quantification, these samples were prepared using the ScheBo^®^ Master Quick-Prep Kit (Schebo Biotech AG, Giessen, Germany) as follows: 4 mg of each stool sample were collected using dipsticks and were diluted and homogenised in 1.2 mL of the buffer provided in the kit to a final concentration of 3.33 mg/mL. After homogenisation, the samples were filtered by a 0.22 µm pore-sized cellulose acetate membrane (FilterBio, Jiangsu, China) and analysed.

Quantification of M2-PK. M2-PK was measured with a commercially available sandwich ELISA M2-PK Stool test (ScheBo^®^ Biotech AG, Giessen, Germany) with monoclonal antibodies against dimeric M2-PK. The stool samples were prepared as previously described and analysed as defined by the manufacturer with a cut-off value of 4 U/mL.

Quantification of TNF-α, IL-6, MMP-2 and MMP-9. Biomarkers were measured in the stool samples using commercial kits (for TNF-α and IL-6) or commercial antibody pairs (for MMP-2 and MMP-9), according to the manufacturer’s specifications, using methods certified on blood adapted by the laboratory to test the stool samples. For that, we have performed a validation assay to evaluate whether the stool matrix itself had an influence on biomarkers determination, and whether these kits/antibodies were able to determine TNF-α, IL-6, MMP-2 and MMP-9 from stools. A specific concentration of the recombinant protein (Table S1) was dissolved in the homogenisation buffer from the ScheBo^®^ Master Quick-Prep Kit (Schebo Biotech AG, Giessen, Germany) and added to the stool samples previously prepared, as described above. These samples were then processed for linearity (parallelism) tests. For this, the stool sample containing the added biomarker (100% concentrated) was serially diluted 1:1 to obtain 50, 25, 12.5, 6.25, and 3.125%. Control samples were prepared without the addition of biomarkers. Then, all samples were subjected to biomarkers’ measurement by using the commercial ELISA kit (for TNF- α and IL-6) or the selected antibody pairs (MMP-2, MMP-9). Detailed information is listed in Table S1. The calibration curves can be found in Supplementary Figure S1. Precision assays were conducted to determine the analytical performance of the optimised ELISA assays by calculating the % of the coefficient of variability (CV) in inter- and intra-assays (Table S2). The % of recovery rate was assessed by spiking a specific amount of each biomarker (according to their dynamic range listed in Table S1) to stool samples (prepared as previously described) and quantifying the biomarkers by the optimised assays (Table S2).

Statistical analysis. Continuous data were presented as mean ± s.d. Kruskal–Wallis test was used to compare biomarker levels of the different groups (negative colonoscopy, other lesions, advanced adenoma, and CRC). Differences were considered statistically significant when *p* ≤ 0.05. Receiver operating characteristic (ROC) curve analysis was performed to determine the overall performance of the biomarkers for CRC detection using the positive group with CRC and the negative group (negative colonoscopy, other lesions, advanced adenoma). Area under the ROC curve (AUC), confidence interval for AUC, sensitivity, and specificity were calculated. We performed a multivariable logistic regression analysis to determine the combined diagnostic accuracy of faecal Hb and M2-PK. We calculated the probability of CRC detection, and we determined the AUC accordingly. Statistical analyses were performed with IBM SPSS Statistics for Windows, Version 22.0. Armonk, NY, USA: IBM Corp.

## 3. Results and Discussion

### 3.1. ELISA Calibration Curves for the Selected Biomarkers

Since the commercial ELISA kits or antibody pairs were not tested for stool samples, we started by optimising the ELISA protocols for these biological samples. The results of the calibration curves for the selected biomarkers in stool samples clearly show a linear correlation between the concentration and the optical density (OD) measurement (Figure S1). Additionally, in the analysis of the precision assays, the % CV of inter- and intra-assay for all biomarkers revealed that the values are within the expected rate for good analytical performance (below 10% for intra-assays and below 15% for inter-assays) (Table S1) Additionally, the recovery rate assay was above 90% for all biomarkers, which is within the optimal values for ELISA assays (Table S2) [29]. This indicates that the stool matrix itself had no influence on MMP-2, MMP-9, TNF-α, and IL-6 determination by ELISA assays, and that the ELISA kits and antibody pairs used were able to determine the selected biomarkers from human stool extracts.

### 3.2. Stool Biomarker’s Levels between Groups

In this study, informed and consenting individuals donated stool samples for analysis, and four groups were defined according to the diagnosis of the individuals, namely patients with CRC, advanced adenoma, other lesions, and controls (Table 1).

**Figure 1:**
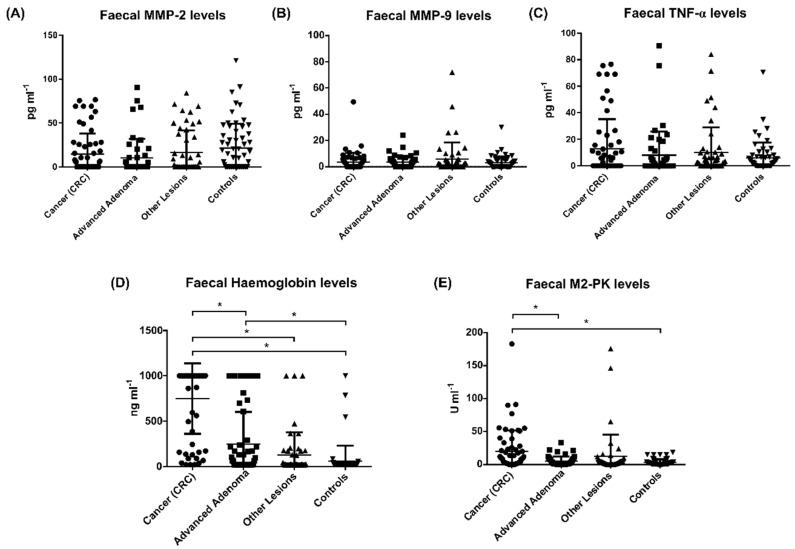
Biomarkers levels in stool samples of the different groups: (**A**) Matrix metalloproteinase (MMP)-2; (**B**) MMP-9; (**C**) Tumour necrosis factor-alpha (TNF-α); (**D**) Haemoglobin (Hb); and (**E**) M2-pyruvate kinase (M2-PK). * Statistically significant differences (*p* ≤ 0.05) of pairwise comparisons between groups.

For assessing the diagnostic value of biomarkers for CRC, the concentration of a panel of biomarkers (MMP-2, MMP-9, TNF-α, M2-PK, and IL-6) was determined in the stool samples by ELISA assays. Additionally, FIT were performed for estimating the concentration of Hb in these samples. The results obtained are presented in Figure 1 and Table 2.

Several studies have described the potential use of MMP-2 and MMP-9 as predictors in colorectal malignancy, since they are over-expressed in tumour tissues, and their levels are increased in serum of individuals with CRC compared to normal controls [30,31]. This could be correlated with increasing Dukes’ stage and poor prognosis in patients with CRC [30,31,32,33,34,35]. Additionally, in stool samples, MMP-9 levels were elevated in CRC patients compared with controls [8,23]. Conversely, in our cohort, we did not find a significant difference in the levels of MMP-2 (Figure 1A) and MMP-9 (Figure 1B) in all four groups of patient samples. In our assays, the concentration of TNF-α in stool samples was very low (mean values of 4–5 pg/mL) and similar for all patient groups (Figure 1C), while IL-6 was not detected in any of the analysed samples. To our knowledge, there are no reports on the detection of these biomarkers in stools from CRC patients, although the serum levels of TNF-α were significantly elevated in individuals with CRC (mainly at stage IV) compared to earlier stages of CRC and controls [26]. Although inflammation is an essential aspect of carcinogenesis, inflammatory molecules typically tend to be produced and act locally. Therefore, detecting low TNF-α levels and not detecting IL-6 in our cohort of stools samples can be due to the local production and consumption of these molecules, not allowing these biomarkers to accumulate in the stools.

As for the Hb quantification (FIT, Figure 1D), there was a significant difference between samples of patients with CRC and all the other groups (advanced adenoma, other lesions, and controls) and between patients with advanced adenoma and the controls. Additionally, as depicted in Figure 1E and Table 2, the median stool M2-PK concentration in individuals with CRC was significantly higher than those of patients with either advanced adenomas or controls. These results are in accordance with other studies that reported increased levels of Hb and M2-PK in stool or blood of patients with CRC or adenomas [7,19,36,37,38].

### 3.3. Determination of the Biomarkers Overall Performance for CRC Detection

**Figure 2:**
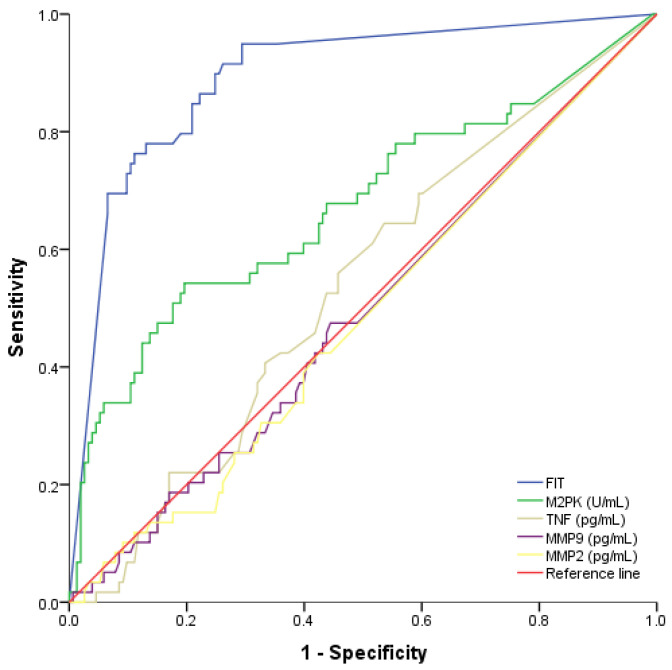
Receiver operating characteristic (ROC) curve for the different biomarkers.

Although the quantification of biomarkers in CRC is essential, calculating the overall performance of a specific biomarker is crucial for the definition of a diagnostic marker. For this, we first evaluated the biomarkers’ cut-off values that determine the clinical sensitivity (ratio of true positives over all individuals with disease) and specificity (ratio of true negatives over all individuals without disease). The cut-off values were defined for Hb on the basis of the available recommendation [39], and for M2-PK, we used the Youden test [40,41] to determine the threshold with the best diagnostic accuracy. Then, we performed a ROC curve analysis (plot of sensitivity versus 1-specificity) on the group of patients with CRC (positive) and the other groups (negative) and determined the AUC that provided a valuable measure for optimal cut-off value selection [42]. The results are presented in Table S3 and Figure 2 and indicate that MMP-2, MMP-9, and TNF-α have no utility for CRC diagnosis with an AUC curve below 0.55. Conversely, M2-PK and Hb showed higher overall performance for CRC with an AUC of around 0.7 and 0.9, respectively.

**Figure 3:**
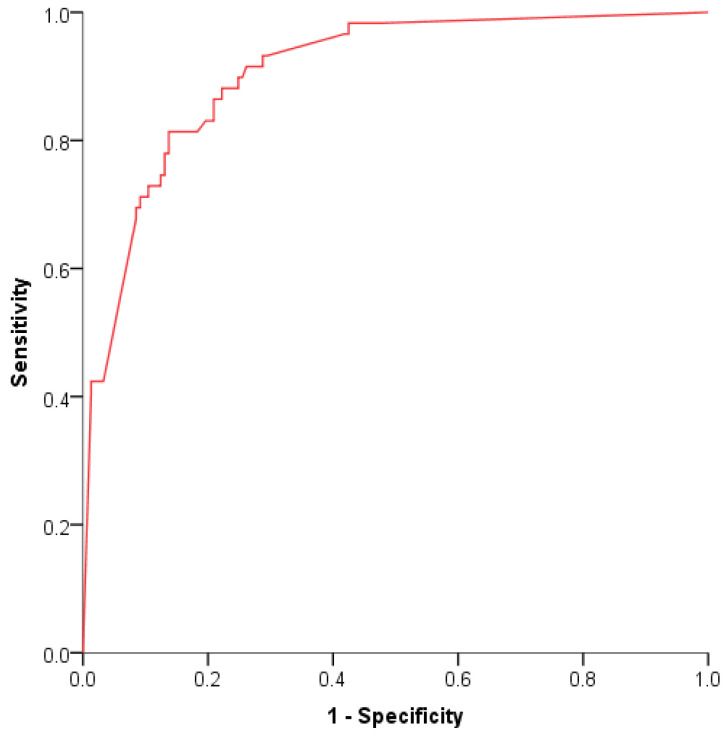
ROC curve for the combination of M2-PK and Hb.

It has been widely accepted that diagnosis based on one single biomarker may not provide sufficient accuracy [43]. Thus, the combination of multiple biomarkers increases the AUC value, thus improving the accuracy of the disease diagnostic tests. Therefore, since Hb and M2-PK were the biomarkers that presented higher AUC for CRC, we further evaluated if the combination of these two biomarkers would increase the sensitivity and specificity of the CRC detection, using the pre-established thresholds. The AUC of the combination of the biomarkers was 0.90. The ROC curve is shown in Figure 3. The results presented in Table 3 indicate that the faecal Hb presented higher sensitivity (92%) than M2-PK (55%) for CRC detection. Sensitivity was increased when either a faecal Hb ≥ 10 µg/g or an M2-PK ≥ 8 U/mL was used to determine a positive result (97%). In contrast, the specificity was increased if a faecal Hb ≥ 10 µg/g and an M2-PK ≥ 8 U/mL was required to determine a positive result (94%). Nevertheless, in both cases, it was not possible to observe an increase in specificity and sensitivity simultaneously. Other studies support the usefulness of a combined screening approach based on the determination of these biomarkers levels in stool samples [13,44]. This can be due to the fact that M2-PK levels in stools enable the detection of both bleeding and non-bleeding tumours, as well as adenomas without false positives originating from other sources of bleeding [7,13,18].

The study herein described had some limitations, namely in the selection of patient samples. In fact, we have used samples from the hospital biobank, and we have performed a diagnostic accuracy analysis. Obviously, the conditions were not daily practice, and we did not include an analysis of the positive and negative predictive value. However, we have performed an analysis of the AUC, sensitivity, specificity, LRs, and diagnostic odds ratio; these values are not modified by the disease prevalence. Additionally, to the best of our knowledge, there are no recent studies showing a difference in the quantitative FITs according to CRC stage [45,46]. In fact, recent meta-analysis [47,48] does not include any information regarding sensitivity and CRC stage. Additionally, although inflammatory biomarkers are not specific, since they are common to different medical conditions, their use in combination with more specific biomarkers for CRC, such as FIT-Hb, can improve diagnostic precision. Overall, it would be interesting in the future to investigate the diagnostic performance of different biomarkers by CRC stage.

## 4. Conclusions

Overall, in our study, we analysed a panel of biomarkers in stool samples and exploited their potential for CRC diagnosis. The results obtained revealed that MMP-2, MMP-9, IL-6, and TNF-α did not offer diagnostic value for CRC. Conversely, the FIT-Hb combined with the determination of M2-PK levels in stool samples may be a means of identifying those individuals at low/high risk of malignant disease and thus help doctors in deciding the need for invasive investigations such as colonoscopy. Further research in larger and more representative populations is required to determine the usefulness of these biomarkers in stratifying both symptomatic and asymptomatic individuals.

The detection of biomarkers in stool is an emerging technology in screening for CRC, since it presents several advantages, such as being non-invasive and simple to use. There are still few studies addressing the identification and quantification of biomarkers in stool samples, and thus further research is of utmost importance to assess the potential of a panel of biomarkers in the detection of CRC. Ideally, a combination of these biomarkers should be implemented in a screening methodology to ensure high sensitivity for CRC detection.

## Figures and Tables

**Table 1:** Clinical Data from Patients and Controls.

Group Description		Cancer (CRC)	Advanced Adenoma	Other Lesions	Controls
Patient Number		60	47	49	60
Mean Age, Range (years)		69, (40–88)	71, (47–86)	65, (17–86)	64, (37–87)
Gender, *n* (%)	Female	23 (38%)	18 (38%)	20 (41%)	38 (63%)
	Male	37 (62%)	29 (62%)	29 (59%)	22 (37%)

**Table 2:** Biomarkers levels in the different groups.

Groups		Hb (ng/mL)	M2-PK (U/mL)	TNF (pg/mL)	MMP-9 (pg/mL)	MMP-2 (pg/mL)
CRC	Number	60.00	60.00	60.00	60.00	60.00
	Mean	748.56	20.21	4.42	3.44	14.63
	Median	1000.00	8.66	3.78	0.00	0.00
	SD	388.86	31.25	4.53	7.11	23.36
Advanced Adenoma	Number	47.00	47.00	47.00	47.00	47.00
	Mean	247.72	5.14	5.31	3.34	10.19
	Median	58.50	1.46	1.86	0.33	0.00
	SD	353.99	7.26	7.57	4.85	21.82
Other lesions	Number	49.00	49.00	49.00	49.00	49.00
	Mean	127.67	12.42	4.24	5.66	16.59
	Median	19.00	2.86	3.40	0.00	0.00
	SD	247.563	33.20	4.89	12.89	24.95
Controls	Number	60.00	60.00	60.00	60.00	60.00
	Mean	59.35	3.56	4.93	3.40	21.58
	Median	19.00	1.68	2.63	1.00	10.25
	SD	171.44	4.44	7.66	4.81	27.64
Total	Number	214.00	214.00	216.00	216.00	216.00
	Mean	305.50	10.56	4.71	3.83	16.04
	Median	23.50	2.78	2.63	0.00	0.00
	SD	411.14	24.03	6.29	7.96	24.84

**Table 3:** Evaluation of the accuracy of the evaluated strategies for significant diagnosis of CRC.

Biomarkers Parameters	Sensitivity (%)	Specificity (%)	LR *+	LR−	Odds Ratio
Hb ≥ 10 µg/g	91.5 (80.6–96.8)	72.3 (64.4–79.0)	3.3 (2.5–4.3)	0.1 (0.05–0.3)	28.1 (10.5–75.0)
M2-PK ≥ 8 U/mL	55.0 (41.7–67.7)	72.4 (65.8–78.2)	2.6 (1.8–3.9)	0.6 (0.4–0.8)	4.7 (5.4–8.8)
Hb ≥ 10 µg/g and M2-PK ≥ 8 U/mL	49.1 (36.0–62.4)	93.5 (88.0–96.6)	7.5 (3.9–14.4)	0.5 (0.4–0.7)	13.8 (6.1–31.4)
Hb ≥ 10 µg/g or M2-PK ≥ 8 U/mL	96.6 (87.2–99.4)	58.2 (49.9–66.0)	2.3 (1.9–2.8)	0.06 (0.01–0.2)	39.6 (9.3–168.3)

* LR—Likelihood ratio.

## Data Availability

Data sharing is not applicable to this article.

## Supplementary Materials

The following are available online at https://www.mdpi.com/article/10.3390/cancers13215568/s1, Figure S1: Calibration curves of the different biomarkers: (**A**) MMP-2; (**B**) MMP-9; (**C**) TNF-α; (**D**) IL-6. Table S1. Conditions used in the ELISA assays for the biomarkers TNFα-, IL-6, MMP-2 and MMP-9; Table S2. Analytical performance of the optimised ELISA assays. Table S3. ROC curves for CRC diagnosis.
