# Supplementary material for: Faecal Diagnostic Biomarkers for Colorectal Cancer
Source: Cancers (Basel). 2021 Nov 7;13(21):5568. doi: 10.3390/cancers13215568 (PMC8582677; doi:10.3390/cancers13215568)
Supplement: Supplementary file 1 [file cancers-13-05568-s001.zip › cancers-1310761-supplementary.pdf]

cancers

MDPI

Article

Diagnostic biomarkers as predictors of colorectal cancer

Andrea Cruz [1*], Carla M. Carvalho [1*], Alexandra Cunha [1], Anais Crespo [2], Águeda Iglesias [2], Laura García-Nimo [3], Paulo P. Freitas [1#] and Joaquín Cubiella [2#,*]

Supplementary Materials:

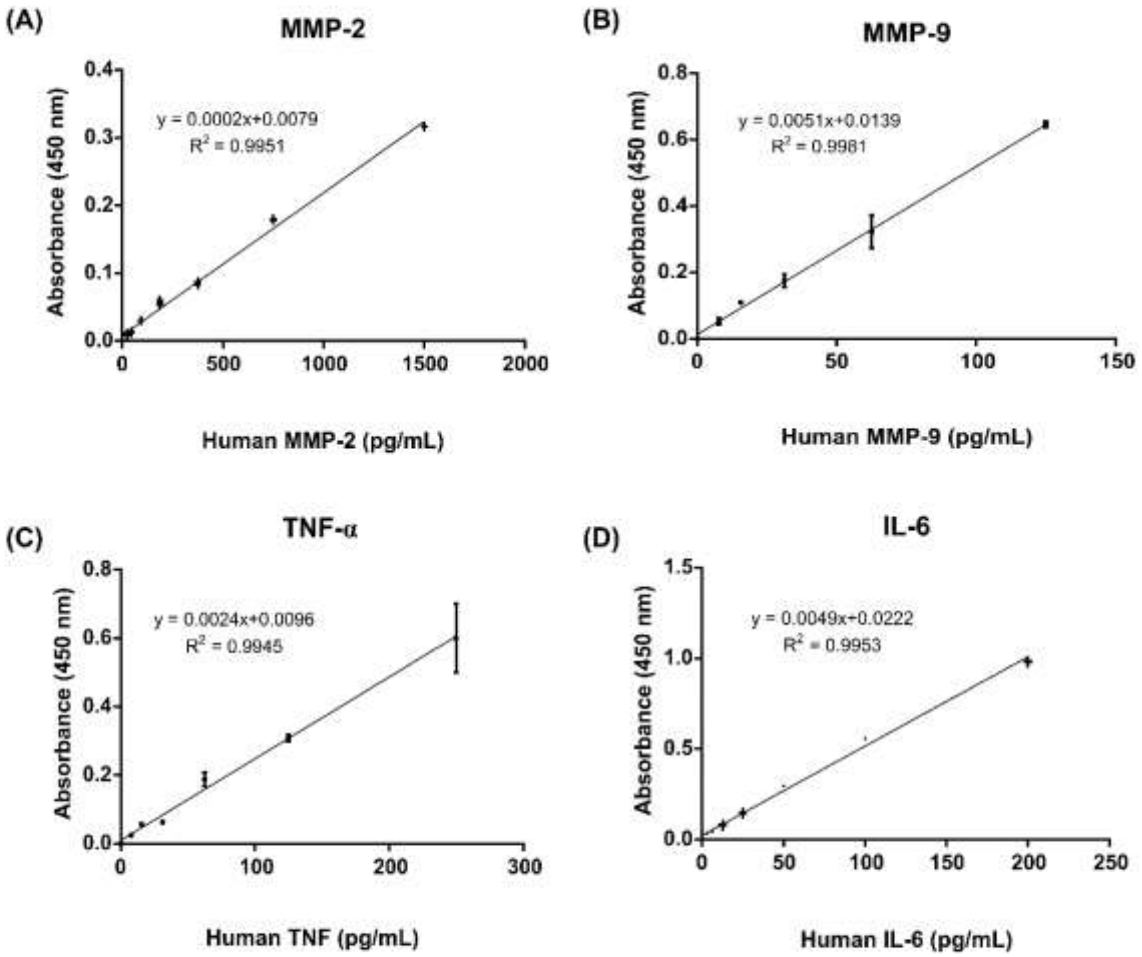

Figure S1. Calibration curves of the different biomarkers: (**A**) MMP-2; (**B**) MMP-9; (**C**) TNF-α; (**D**) IL-6.

Table S1. Conditions used in the ELISA assays for the biomarkers TNFα-, IL-6, MMP-2 and MMP-9.

	Biomarker	Recombinant Protein Concentration	Linear range	ELISA/Antibody information
TNF-α	Human recombinant TNF (Cat. No.: 88-7346-22, ebioscience)	250 pg/mL	4–250 pg/mL	Human TNF-α Elisa Kit (Cat # 88-7346-88, Invitrogen)
IL-6	Human recombinant IL-6 (Cat. No.: 88-7066-88, ebioscience)	200 pg/mL	3–200 pg/mL	Human IL-6 Elisa Kit (Cat # 88-7066-22, Invitrogen)
MMP-2	Human recombinant MMP-2 (Part 76963, Biolegend)	1500 pg/mL	23–1500 pg/mL	Purified anti-MMP-2 (Clone: M6303D01, 0.5 mg/mL, Biolegend); 2 μg/mL<br>Biotin anti-human MMP-2 (Clone: M6310C11, 0.5 mg/mL, Biolegend); 1 μg/mL
MMP-9	Human Recombinant MMP-9 (Met1-Asp707) Protein (Cat No.: 1399990243, Immunostep)	125 pg/mL	7.8–125 pg/mL	Anti-Human MMP-9 purified mouse monoclonal (REF. 1399990241, 1 mg/mL, Immunostep); 4 μg/mL<br>Anti-Human MMP-9 Biotin Rabbit Polyclonal (REF. 1399990242, 0.77 mg/mL, Immunostep); 2 μg/mL

Table S2. Analytical performance of the optimized ELISA assays.

Biomarker	Inter-Assay (CV%) (n = 8)	Intra-Assay (CV%) (n = 100)	% Recovery rate (n = 10)
TNF (pg/mL)	9.6	9.33	98.9± 5.5
IL-6 (pg/mL)	8.62	2.81*	92.2± 4.1
MMP-9 (pg/mL)	4.98	6.97	93.5% ± 1.79
MMP-2 (pg/mL)	6.32	5.79	100.1% ± 1.0

Note: Inter-assay (CV%) was determined by measuring ELISA controls with low and high concentrations in multiple assays to monitor plate-to-plate variations; Intra-assay (%CV) was determined to analyse the variation of triplicates of samples and ELISA controls; % Recover rate –was determined to assess the reliability of the methodology. * For IL-6, the n = 10 and was calculated using the data from the ELISA controls, since we did not detect this biomarker in the samples.

Table S3. ROC curves for CRC diagnosis.

Contrast Result Variables	Area Under the Curve	Error Tip.
Hb (ng/mL)	0.892	0.025
M2-PK (U/mL)	0.673	0.045
TNF (pg/mL)	0.535	0.043
MMP-9 (pg/mL)	0.489	0.044
MMP-2 (pg/mL)	0.481	0.044
